# Water, Sanitation, and Hygiene Facilities and Hygiene Practices Associated with Diarrhea and Vomiting in Monastic Schools, Myanmar

**DOI:** 10.4269/ajtmh.15-0290

**Published:** 2016-08-03

**Authors:** Emma R. N. Weaver, Paul A. Agius, Hilary Veale, Karl Dorning, Thein T. Hlang, Poe P. Aung, Freya J. I. Fowkes, Margaret E. Hellard

**Affiliations:** ^1^ Centre for Population Health, Burnet Institute, Melbourne, Australia; ^2^ Burnet Institute Myanmar, Yangon, Myanmar; ^3^ Department of Epidemiology and Preventive Medicine, Monash University, Melbourne, Australia; ^4^ Melbourne School of Population and Global Health, Centre for Epidemiology and Biostatistics, The University of Melbourne, Melbourne, Australia; ^5^ Department of Infectious Diseases, Monash University, Melbourne, Australia

## Abstract

Gastrointestinal diseases are major contributors to mortality among children globally, causing one in 10 child deaths. Although most deaths are in children aged ≤ 5 years, the burden of disease in school-aged children is still considerable and contributes to high rates of school absenteeism. This study investigates behavioral and structural risk factors associated with diarrhea and/or vomiting among schoolchildren in Myanmar. Cross-sectional data from a school-based multistage cluster sample of grade 4 and 5 students were analyzed to explore water, sanitation, and hygiene (WASH) facilities and hygiene-related practices of students in monastic schools in Myanmar. The outcome of interest was student self-reported diarrhea and/or vomiting in the past week. Random effects multinomial logistic regression models were used to explore correlates at the student and school level. A total of 2,082 students from 116 schools across eight states/regions were included. Of these, 11% (223) self-reported at least one episode of diarrhea only, 12% (253) at least one episode of vomiting only, and 12% (244) diarrhea and vomiting in the past week. Independent risk factors associated with the outcome included poor availability of handwash stations, no access to a septic tank toilet, inconsistent toilet use, and lower student grade. These findings highlight the importance of having an adequate number of handwash stations for students, the provision of septic tank toilets, and consistent toilet use. Future WASH programs need to target not only the provision of these WASH facilities but also their utilization, particularly among younger school-aged children.

## Introduction

Gastrointestinal diseases are major contributors to mortality among children globally, causing one in 10 child deaths.^1^ Although most deaths are in children aged ≤ 5 years, the burden of disease in school-aged children is still considerable and contributes to high rates of school absenteeism.^2^,^3^ Water, sanitation, and hygiene (WASH) interventions aim to increase access to basic necessities, including clean water, safe stool disposal, and food sources free from contamination, and in doing so, break the fecal–oral route of transmission to reduce gastroenteritis. Prevention measures for diarrheal transmission include primary barriers—safe stool disposal and the removal of fecal matter from hands—and secondary barriers—good handwashing before preparing food, cooking, and eating.^4^,^5^ WASH interventions target the provision of sanitation facilities to ensure safe stool disposal,^6^ quality drinking water supply,^7^ handwashing facilities, and behavior change techniques to promote effective use of toilets, handwashing behavior,^8^ and general hygiene practices.

The impact of WASH interventions on mitigating illness have been well documented.^6^,^9^ A systematic review found that interventions that promoted both handwashing after defecation (a primary barrier) and washing hands before handling food (a secondary barrier), resulted in a 47% reduction in diarrheal risk.^9^ Two studies found that interrupting the primary barriers to pathogen transmission, such as safe stool disposal, is more important than interrupting the secondary barriers, such as good handwashing before eating.^5^,^10^ A recent meta-analysis concluded that hygiene, sanitation, water supply, and water quality interventions can all reduce illness, particularly interventions to improve the microbial quality of drinking water.^7^ However, multiple interventions (consisting of combined WASH measures) were no more effective than interventions with a single focus.^7^ Furthermore, hygiene promotion studies have provided little reliable evidence on the effectiveness of interventions aimed at changing behavior.^11^

The impact of school-based WASH interventions on school-aged children is also unclear.^12^ Similar studies exploring the impact of the provision of water on handwashing in schools found different results—in Israel there were no significant reductions in illness or school absenteeism, whereas in Egypt and China there were significant reductions in rates of illness.^13^–^15^ These differences are most likely due to variation in the prevalence of target illnesses between particular regions, issues with confounding that are best controlled for using blinding, and critical pathways for gastrointestinal disease being highly context specific.^12^,^16^,^17^

In Myanmar, gastrointestinal diseases are a leading cause of morbidity and mortality among children.^18^ Monastic schools currently operate across the country, hosting over 269,000 children.^19^ Monastic schools receive very little government funding, are run by monks, generally do not charge tuition fees, and serve the poorest children in the country.^20^ This population is at particularly high risk of gastrointestinal diseases because of their low socioeconomic background and the conditions within many of these schools. The aim of this study was to investigate risk factors associated with diarrhea and vomiting among these school-based children before the implementation of a WASH program. Data were collected on a range of behavioral and resource-specific factors, both at the student and school levels, with the view to exploring whether particular behavioral or structural factors placed students at greater risk of self-reported diarrhea and vomiting.

## Materials and Methods

### Study design.

Cross-sectional data from a school-based multistage cluster sample of grade 4 and 5 students were analyzed to explore WASH facilities and hygiene-related practices of students in monastic schools in Myanmar. This research was part of a larger program entitled “Building the capacity of the monastic school system in Myanmar” and aimed to improve school management, teaching practices, the school environment, school hygiene practices, and the level of community support at monastery schools throughout Myanmar.^20^ This study received ethical approval from the Alfred Hospital, Melbourne, Australia, and the Department of Medical Research, Lower Myanmar.

### Sampling.

**Figure 1.**
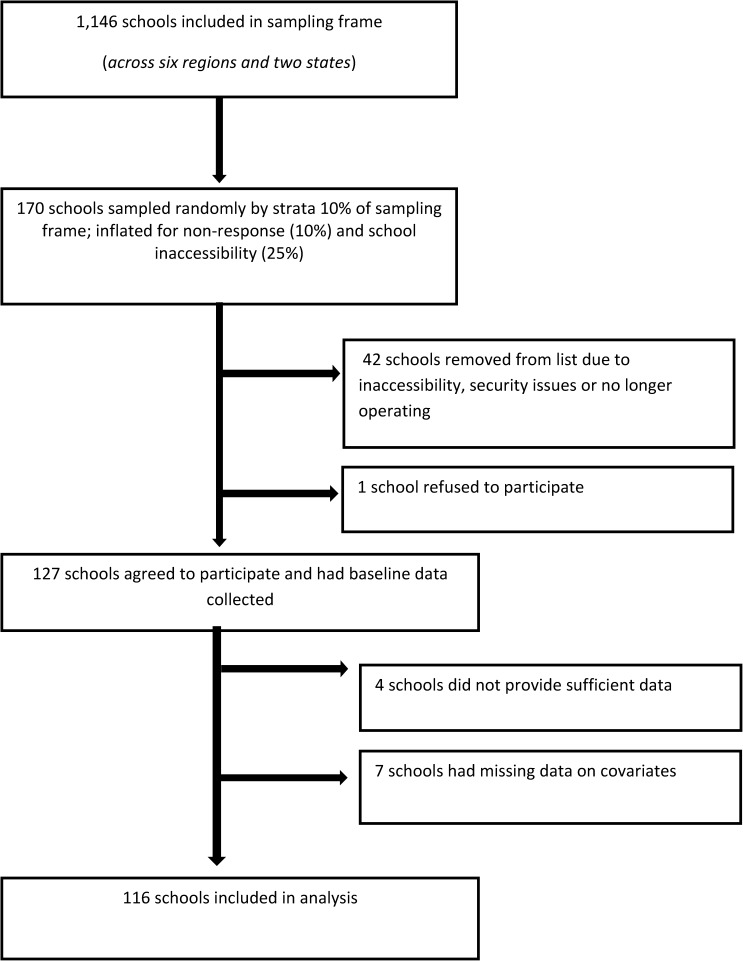
Flow chart of recruitment of monastic schools.

Given the resource limitations and locational complexities of the study, sampling of schools for the baseline WASH study focused on eight regional areas of Myanmar that were thought to be representative, sociodemographically and geographically, of monastery schools in Myanmar. Some states and regions were excluded from the sampling frame because of security concerns and access difficulties. The sampling frame for this study comprised 1,146 schools across six regions and two states as shown in Figure 1

and Table 1.

Sampling comprised two stages: at the first, schools were randomly selected using a proportional allocation sampling fraction of 10%, and at the second, (approximately) 10 students from each target grade (4 and 5) of the selected school were randomly selected to participate in the study (Figure 1). In terms of exploratory cross-sectional research, the sampling fraction was a balance between having a target sample that was achievable given the scope of the study and its logistical resources, and ensuring sampling was sizeable enough to have an acceptable standard error in prevalence estimation. We estimated that, given the design effect from the sampling approach (assuming a within-school intraclass correlation [ICC] = 0.05) a target sample of *N* = 115 schools (sampling fraction 10%) would enable us to report prevalence with an approximate margin of error (95%) of 3.5%. To improve the representativeness of the sample and reduce the variance in point estimation, schools were sampled proportionately across regional strata using the 10% sampling fraction. The target sample was inflated by 10% to account for school nonresponse, and a further 25% for refusal and inaccessibility. A total of 170 schools were randomly selected from eight regional strata. From this inflated sample, 42 schools did not participate because of inaccessibility, security reasons, and school closures, and one school refused to participate. One hundred and twenty-seven schools agreed to participate in the study (participation rate 75%), yielding a total sample of 2,256 students.

### Data collection.

Quantitative data collection tools were developed in English and translated into Burmese, which included a self-administered student questionnaire and a school environment observation checklist completed by local fieldworkers. A self-administered structured student questionnaire was used as this was considered the most efficient data collection method given logistical constraints, the size of the target sample, and the relatively short period allocated for data collection. These survey instruments were piloted by five fieldworkers at two schools and were subsequently refined. Fieldworkers met with principals at schools selected to participate in the WASH baseline study. They explained the WASH study and survey tools and asked for permission to conduct the study. A monetary reimbursement for the principal's time was offered. Five teams of four fieldworkers completed school recruitment and data collection. Data collection was conducted in two phases—July–August 2013 (80 schools in three regions) and September–November 2013 (47 schools in the remaining regions and states).

The school environment checklist was completed by fieldworkers with assistance from a school staff member. Photos were taken by fieldworkers to accurately record key features of the school environment and facilities. For the self-administered student questionnaire, a simple explanation of the instrument and study was given by fieldworkers to the students, followed by an invitation to participate in the survey. Questionnaires were completed away from other students. Approximately 20 minutes were allocated to students to complete the questionnaire. Fieldworkers offered assistance to students if required, for example, explaining the meaning of questions and how to record answers on the instrument.

### Data management.

The student questionnaires and school environment observation data were entered by fieldworkers into a relationally organized EpiData database (EpiData Association, Odense, Denmark). These relational data were linked using key matching data and prepared for analysis using Stata, version 12 (StataCorp, College Station, TX).

### Measures.

#### Outcome.

##### Diarrhea and vomiting.

The outcomes of interest in this study were student self-reported diarrhea and/or vomiting in the past week, with the measure derived from two separate questions pertaining to these symptoms. Students were asked, “In the past week, have you had diarrhea?” where diarrhea was defined as “more than three loose bowel movements in one day.” Similarly, students were asked, “In the past week, have you had vomiting?” For both questions, students could respond: “no,” “just on one day,” or “on more than one day.” For analysis, student responses to these questions were rescored to form a polytomous measure of experience of diarrhea and vomiting, where students were categorized as experiencing in the past week an episode of “diarrhea only,” “vomiting only,” “both diarrhea and vomiting,” or “no episode of either.”

To assess sanitation and hygiene-related risk, both behavioral and school environment factors were measured.

#### Hygiene practices.

##### Handwashing.

Participants were asked to report the frequency of their handwashing practice before meals and after toilet use using an ordinal measure: “never,” “sometimes,” “mostly,” and “always.” For analysis student responses were collapsed into a binary measure whereby “never” and “sometimes” was treated as “poor,” and “mostly” and “always” treated as “good” handwashing behavior.

##### Toilet use.

Participants use of toilets was measured by asking students if they used the school toilet “always,” “some of the time,” or “never.” For analysis, this factor was then collapsed into a binary measure whereby students responding “always” were compared with those responding “some of the time” or “never.”

##### Diarrhea prevented student from attending class.

Participants were asked whether their most recent episode of diarrhea had prevented them from attending class, and asked to respond either “yes” or “no.”

##### Hygiene education recall.

Participants were asked whether they could recall any kind of school lesson about hygiene or cleanliness and could respond to this question with “yes” or “no.”

#### Student hygiene facilities at school.

##### Number of toilets.

The number of student toilets was recorded at each school. For analysis, using sample tertiles, students were rescored into three ordinal groups representing the number of toilets students had access to at school.

##### Student-to-facility ratios.

Student-to-facility ratios were coded as 3-level ordinal factors based on the recommended United Nations Children's Emergency Fund/World Health Organization (WHO) guidelines for boys (handwash station ratio: ≤ 50:1, 51:1–150:1, and ≥ 151:1 and toilet ratio: ≤ 50:1, 51:1–100:1, and ≥ 101:1).^21^

##### Type of toilet.

Type of toilet was a multiple response measure, where schools could be coded as having “pour flush,” “pit latrine,” and “septic tank” toilet arrangements.

##### Cleanliness of student toilets.

The cleanliness of student toilets was a nominal measure, where school toilets were coded as “clean,” “somewhat clean,” and “not clean.” These data were then rescored to a binary measure comparing “clean” versus “unclean” (“somewhat clean” and “not clean”) toilets.

##### No separate toilets for girls.

The provision of a separate toilet for female students was treated as a binary measure.

##### Number of handwash stations.

The number of handwash stations for students was recorded at each school. For analysis, using sample tertiles, students were rescored into three ordinal groups representing the number of handwash stations students had access to at school.

##### Location of handwash stations.

The location of handwash stations was measured by a nominal measure “near to toilets,” “inside toilet compartment,” “in/near building,” and “other.” For analyses these data were collapsed into a binary measure of proximity to toilet location (“near in toilet building” versus “far from toilets.”

##### Insufficient soap and water at handwash stations.

Insufficient soap and insufficient water were binary measures, where a school was considered to have insufficient water if water was evident at fewer than 50% of handwash facilities and insufficient soap if soap was evident at fewer than 50% of facilities. Students were considered to have insufficient soap and/or water if a school did not have handwashing facilities.

##### Unimproved water storage.

Water storage was a nominal measure where schools were coded to have “uncovered pot,” “covered pot,” “tank with tap,” “filter pot with tap,” “piped water with tap,” “bottled water,” or “direct from tube well” water storage. On the basis of the WHO guidelines,^22^ these data were rescored to a binary measure of unimproved water storage versus improved water storage, with improved water storage defined as covered pot, tank with tap, bottled water, filter pot with tap, piped, or direct from tube well storage.

### Analysis.

Contingency table analyses were used to provide prevalence estimates of diarrhea and vomiting. In univariable analyses, standard errors and associated 95% confidence intervals (CI) around prevalence estimates took account of the inherent clustering and stratification in sampling.^23^ Generalized linear latent and mixed modeling (gllamm)^24^ was used to estimate random effect multinomial logistic regression (i.e., specifying a multinomial link function and binomial distribution) models, exploring correlates of experience of diarrhea, where school was treated as a random intercept and student and school level covariates estimated as fixed factors. In multinomial regression models, the outcome reference group was of students who had not experienced either diarrhea or vomiting in the past week. Seemingly unrelated estimation^25^ was undertaken to test that the multinomial regression model met the independence of irrelevant alternatives (IIA) assumption.^26^ Random effect multivariable multinomial regression analyses treated school as a latent factor, and therefore modeled the dependency in school clusters directly, providing effect estimates for study exposures, which took account of school-specific variance in rates of diarrhea and vomiting (i.e., between-school heterogeneity in the outcome induced by unobserved factors). Where differences in effect (both in direction and magnitude) were observed across outcome responses, post-estimation Wald tests were conducted to test the differences in effect of each exposure across outcome responses. A complete case approach to analyses was undertaken. All statistical analyses were conducted using the Stata statistical software package, version 13 (StataCorp).

The key risk factors included in the analyses were handwashing practice before meals and after toilet use, toilet use, hygiene education recall, number of student toilets at the school, number of handwash stations for students at the school, cleanliness of student toilets, no separate toilets for girls, insufficient soap at handwash stations, insufficient water at handwash stations, type of toilet, and presence of unimproved water storage at the school. Student sex and grade were also included in multivariable models. In addition to the number of student toilets at the school and the number of handwash stations for students, for comparative purposes, adjusted analyses of the association between student-to-facility ratio measures and diarrhea and/or vomiting were undertaken and are shown in Supplemental Table 1.

## Results

The study sample comprised 2,256 students from 127 monastic schools. Of these, 174 students (8%) were excluded—67 because of nonresponse on both the outcome (*N* = 5, 3%) and model covariates (*N* = 62, 37%), and a further 107 (60%) because of their attendance at schools with no toilets—given we modeled a range of factors relating to toilet quality. The remaining analysis was based on 2,082 students from 116 schools with at least one student toilet (Table 1). Analysis of missing data showed no significant differences in the proportion of students reporting diarrhea and/or vomiting between cases included and excluded from analyses (diarrhea: 12% versus 11%, vomiting: 12% versus 14%, diarrhea and vomiting: 12% versus 11%; joint Wald test: χ^2^(3) = 0.94, *P* = 0.81). Further, comparison of rates of diarrhea and vomiting between those with and without toilets showed no significant differences (diarrhea: 12% versus 11%; vomiting: 12% versus 16%; diarrhea and vomiting: 11% versus 8%; joint Wald test: χ^2^(3) = 2.6, *P* = 0.46).

### Demographic factors and hygiene practices.

Of those included in the analysis, 50% of students were female (*N* = 1,030), 50% were in grade 5 (*N* = 1,049), and the median age was 10 years (interquartile range: 9–11; Table 2). When asked about school toilet use, 51% (*N* = 1,060) of students said they did not always use the toilet. Of those who reported not using the toilet, most attributed their nonuse to the condition of the toilet (*N* = 483, 46%) or poor accessibility (*N* = 359, 34%).

### Student hygiene facilities at school.

Twenty per cent (*N* = 23) of schools did not provide handwash station facilities to students. Of schools with at least one handwash station (*N* = 93), 89% (*N* = 83) had handwash stations with insufficient soap and 32% (*N* = 30) had insufficient water (Table 2). The location of handwash stations varied, with 71% (*N* = 66) of schools not having handwash stations near the toilets.

Of schools with toilets, 48% (*N* = 55) had a student-to-toilet ratio of ≤ 50:1, 37% (*N* = 43) had 51:1–100:1, and 17% (*N* = 17) had > 100:1. For one school, no information was collected regarding the total student population at the school, and so it was excluded from this ratio. Of schools with handwash station, 37% (*N* = 33) had a student-to-handwash station ratio of ≤ 50:1, 40% (*N* = 36) had 51:1–150:1, and 23% (*N* = 20) had > 150:1. Twenty-three schools had no handwashing stations and were excluded from ratio estimates. Multinomial regression analyses showed no significant unadjusted association (Supplemental Table 1 shows adjusted associations) between both student-to-toilet ratio and experience of diarrhea and/or vomiting (joint Wald test: χ^2^(6) = 6.8, *P* = 0.34) and student-to-handwash station ratio and experience of diarrhea and/or vomiting (joint Wald test: χ^2^(6) = 3.8, *P* = 0.71).

### Factors associated with diarrhea and vomiting.

Univariable and multivariable analyses exploring the correlates of diarrhea and vomiting are shown in Tables 3 and 4. In multivariable analysis, compared with those in grade 5, students in grade 4 were significantly more likely to report episodes of diarrhea only (adjusted relative risk ratio [ARR] = 1.40, 95% CI = 1.05–1.86, *P* = 0.02), vomiting only (ARR = 1.75, 95% CI = 1.32–2.33, *P* < 0.01), and both diarrhea and vomiting (ARR = 1.48, 95% CI = 1.10–1.99, *P* = 0.01) in the past week. A post-estimation Wald test showed that this higher risk for grade 4 students was consistent across illness outcomes (Wald test: χ^2^(2) = 1.67, *P* = 0.43). Poor handwashing before meals was also associated with higher risk of illness. Students who reported poor handwashing before meals were 43% more likely to report an episode of vomiting only (ARR = 1.43, 95% CI = 1.00–2.05, *P* = 0.05). Higher risk was also observed with diarrhea and vomiting (ARR = 1.31, 95% CI = 0.90–1.91, *P* = 0.16) and diarrhea-only (ARR = 1.06, 95% CI = 0.72–1.57, *P* = 0.76) episodes but with a smaller magnitude of effect and was not statistically significant.

Not using the toilet consistently was also associated with higher risk of illness. Students reporting no or inconsistent use of the school toilet were 55% more likely to report an episode of both diarrhea and vomiting in the past week (ARR = 1.55, 95% CI = 1.12–2.14, *P* = 0.01) and vomiting only (ARR = 1.31, 95% CI = 0.96–1.80, *P* = 0.09) compared with students who “always” used the school toilet. Higher risk was also observed for diarrhea-only episodes but this was not statistically significant (ARR = 1.08, 95% CI = 0.79–1.48, *P* = 0.64).

Students at schools with septic tank toilets were less likely to report diarrhea and vomiting (ARR = 0.66, 95% CI = 0.42–1.05, *P* = 0.08) and diarrhea only (ARR = 0.61, 95% CI = 0.39–0.96, *P* = 0.03) in the past week compared with students at schools without this type of toilet. Lower risk was also observed for vomiting-only episodes but this was not statistically significant (ARR = 0.80, 95% CI = 0.51–1.25, *P* = 0.33). A post-estimation Wald test showed that this lower risk for students at schools with septic tank toilets was consistent across illness outcomes (Wald test: χ^2^(2) = 1.98, *P* = 0.37).

Students from schools with little or no access to handwash stations (either none or one handwash station) were almost twice as likely to self-report diarrhea-only (ARR = 1.85, 95% CI = 0.98–3.49, *P* = 0.06) and vomiting-only (ARR = 2.02, 95% CI = 1.05–3.87, *P* = 0.03) episodes in the past week than students at schools with the highest number of handwash stations (between four and 20). Although the same higher risk was observed for diarrhea and vomiting, the increase in risk from having relatively few (lowest group) handwash stations was smaller in magnitude (67% higher risk) and not statistically significant (ARR = 1.61, 95% CI = 0.84–3.09, *P* = 0.12). A post-estimation Wald test showed that this higher risk for students at schools with few handwash stations was consistent across illness outcomes (Wald test: χ^2^(2) = 0.63, *P* = 0.43). Conversely, insufficient water at handwash stations was associated with a lower risk (∼55%) of diarrhea-only (ARR = 0.45, 95% CI = 0.22–0.91, *P* = 0.03) and vomiting-only (ARR = 0.56, 95% CI = 0.28–1.09, *P* = 0.09—marginally nonsignificant) episodes, and a statistically nonsignificant 47% increase in risk for diarrhea and vomiting (ARR = 1.47, 95% CI = 0.75–2.89, *P* = 0.26). Further, a post-estimation Wald test showed that the observed difference in effect of insufficient water at handwash stations across the response outcomes was statistically inconsistent across illness outcomes (Wald test: χ^2^(2) = 15.99, *P* < 0.01); perhaps pointing toward unmeasured factors—likely at the school level—which may account for this counterintuitive association.

Surprisingly, students at schools with a moderate number of toilets (three to five) were 51% less likely to self-report diarrhea and vomiting than students at schools with the highest number of toilets (6–20; ARR = 0.49, 95% CI = 0.27–0.89, *P* = 0.01). Reduced risk was also observed for diarrhea-only and vomiting-only episodes, but was smaller in magnitude and not statistically significant (diarrhea only: ARR = 0.74, 95% CI = 0.42–1.31, *P* = 0.31; vomiting only: ARR = 0.90, 95% CI = 0.51–1.61, *P* = 0.73). A post-estimation Wald test showed that for students attending a school with a moderate number of toilets, the estimated risk was inconsistent across illness outcomes (Wald test: χ^2^(2) = 5.45, *P* = 0.02), again suggesting potential unmeasured confounding in modeling.

In multivariable analysis, poor handwashing after toilet use, insufficient soap, unclean toilets, availability of pour flush or pit latrine toilets, and unimproved water storage at the school were not significantly associated with experience of diarrhea/vomiting in the past week. Given the model covariates, the conditional ICC—in effect indicating the level of between-school heterogeneity in diarrhea and vomiting—was small to moderate (*ρ* = 0.18). Finally, seemingly unrelated estimation on alternate outcome multinomial models indicated that the specified multivariable model met the IIA assumption (Wald test: χ^2^(108) = 98.8, *P* = 0.73).

## Discussion

Diarrhea and vomiting was common in our sample with over one-third of students self-reporting diarrhea, vomiting or diarrhea, and vomiting in the past week. Although direct comparisons of disease frequencies are difficult because of variations in recall period, this proportion is higher than diarrheal episodes reported per week in similar school-aged children in other studies in developing countries (2–22%).^27^–^29^

Our results suggest that the poor availability of handwash stations in schools increases the risk of students self-reporting diarrhea- and vomiting-only episodes in the past week. Where fieldworkers observed only one or no handwash stations, students were significantly more likely to self-report both these outcomes. The number of handwash stations at schools was shown to be associated with vomiting and/or diarrhea in our analysis, suggesting that having easily accessible hygiene facilities is important to reduce diarrhea and vomiting. The availability of septic tank toilets was also associated with reduced risk of diarrhea and vomiting among students. Interestingly, fewer available toilets in schools was not associated with an increase in diarrhea or vomiting among students, independent of other factors. This counterintuitive finding suggests there are unmeasured factors at the school level that confound the results presented here.

Our findings also suggest that, regardless of the number of available toilets, not using the school toilet consistently heightened students' risk of diarrhea and vomiting. This suggests that despite their condition, it is worth encouraging students to use toilets as opposed to alternative sanitation practices, like using a nearby field or not using the toilet during school hours.

The self-reported rate of handwashing before meals and after toilet use was high in our study, with over three-quarters of students reporting “always” or “mostly” washing their hands on these occasions. These findings are notably higher than other studies; a review by The Global Public-Private Partnership for Handwashing, including studies using observational measures for handwashing practices from Asia and Africa, found rates for handwashing after toilet use and before meals ranging from 3% to 42% and 1% to 16%, respectively.^30^ Our results may therefore reflect the way in which data were collected, that is, through a self-reported structured questionnaire as opposed to observation. Self-report can overestimate rates of handwashing, sometimes by 2- or 3-fold.^16^,^31^ Although an association was observed between poor handwashing before meals and higher risk of vomiting-only episodes, the lack of association between handwashing behavior and all three outcomes in our analysis need to be interpreted with caution. There is strong evidence to suggest that improving handwashing behavior is one of the most cost-effective ways to prevent fecal–oral transmission both in developing and developed countries.^27^,^32^,^33^ A meta-analysis by Curtis and others found that interventions to promote handwashing with soap, where compliance was assessed through observation, were associated with a 47% reduction in risk of diarrhea-related infection.^9^

No association was found between schools with unimproved water storage sources, such as uncovered water pot, and self-reported diarrhea or vomiting. This may be because water quality is less important than other transmission routes for diarrheal disease. This finding is consistent with a Filipino study that observed there was little difference between illness rates of children drinking good quality water and those drinking moderately contaminated water.^34^ Similarly, the counterintuitive finding that insufficient water at handwash stations reduced the risk of diarrhea- and vomiting-only episodes suggests there may be unmeasured factors particularly at the school level that confound the relationship between sufficient water supply at handwash facilities and risk of diarrheal disease. This finding again highlights the complexity of pinpointing risk factors within the WASH sector given their overlapping nature.

Students in grade 4 were significantly more likely to self-report all three illness outcomes (diarrhea only, vomiting only, and diarrhea and vomiting) than their grade 5 counterparts in both the univariable and multivariable analyses. The reason for this is not clear, but it may reflect decreased immunity in the younger children or a lower level of maturity and responsibility to actively maintain good hygiene as has been suggested in other studies.^35^ In this study, however, the association is unlikely attributable to health education, as multivariable analyses were adjusted for hygiene education recall. Regardless of the cause, this finding highlights a need for research to explore the motivations of children at different ages and maturity levels to adopt hygienic handwashing and toilet practices.

The main strengths of our study are that the prevalence and effect estimations were based on a multistage probability sample of grade 4 and 5 students at monastic schools in eight states/regions across Myanmar and that the participation rate was relatively high. Also the analytical methodology adopted in estimating the effect of key risk factors took account of variance in student diarrhea and vomiting rates across schools. However, the study does have some limitations. Some schools were excluded from the sampling frame because of inaccessibility and security concerns, and despite substitution with a randomly selected replacement school, exclusion of difficult-to-reach schools from our sample may introduce some bias to results. Data collection was conducted in two phases for logistical reasons, including the inaccessibility of some schools during the monsoon period and our small number of fieldworkers. This may introduce confounding due to the likely higher risk of diarrhea during the monsoon period of May–September.^36^ Although using student self-administered questionnaires may have resulted in some responder bias including overreporting of good personal hygiene practices,^32^ this data collection approach was restricted to measurement of student behavior only. Measurement of the frequency of student diarrhea and vomiting (“on one day” or “on more than one day”) was limited in this study, and rescoring these data to a nominal measure of experience meant severity of illness was largely unknown. Although our analysis does allow for the estimation of the independent effects of student access to multiple toilet types, we did not have data pertaining to the number of toilets by toilet type at a school, and this prevented an analysis that was sensitive to the extent to which schools were reliant on particular combinations of differing toilet arrangements. This study was cross-sectional and did not capture changes in diarrhea-related infections and factors thought to influence infection over time and during different seasons; therefore, the reader should be mindful that these study results provide no evidence in terms of causality. In addition, although the generalized random effect regression models we used do take account of variance in risk of student diarrhea and vomiting across schools induced by unobserved factors at the school level, given that the random effects model assumes latent unobserved factors are independent of the vector of observed variables estimated in modeling, there is nonetheless the potential for unobserved confounding. Furthermore, we did not measure socioeconomic and family-based risk factors for students outside of the school setting, which may introduce additional unobserved confounding.

Diarrheal diseases are one of the greatest public health burdens affecting resource-poor communities in developing countries.^17^,^37^,^38^ While diarrhea and/or vomiting are only crude indicators of more severe gastroenteritis infection,^39^ our findings highlight the likely benefit of WASH interventions within schools in low-income communities in developing countries. This is further supported by evidence promoting interventions within schools as beneficial to the wider community as students become active change agents within their community.^27^,^28^ Based on our findings, we have identified three priority areas to reduce diarrheal disease in school-aged children in these settings. These include ensuring the adequate provision of well-maintained handwash stations, use of toilets with a septic tank arrangement to manage waste, and encouraging consistent toilet use. It is also recommended that future WASH programs in these settings target not only the provision of these WASH supplies but their utilization, particularly among younger school-aged children.

## Supplementary Material

Supplemental Table.

## Figures and Tables

**Table 1:** Schools included in analysis by region (*N* = 116)

Region	Schools	Students
Metropolitan		
Yangon	19	369
Mandalay	33	591
Plain area		
Bago	12	221
Sagaing	22	389
Hilly		
Chin*	2	28
Shan*	7	119
Coastal		
Ayarwaddy	16	268
Thanintharyi	5	97
All areas	116	2,082

* State not region.

**Table 2:** Sample descriptives showing student demographic and hygiene-related behavioral factors (*N* = 2,082) and school (*N* = 116) hygiene- and sanitation-related structural factors

	*n*	%	95% CI*
Student factors (*N* = 2,082)			
Sex (observed)			
Males	1,052	50.5	47.0–52.0
Grade (observed)			
4	1,033	49.6	47.7–51.6
Age (self-reported)			
Median (IQR)	10 (9–11)	–	–
Hygiene behavior (self-reported)			
Poor handwashing before meals	436	20.9	18.0–24.2
Poor handwashing after toilet use	436	20.9	18.2–24.0
Inconsistent toilet use	1,060	50.9	45.7–56.1
Diarrhea prevented student from attending class	863	41.5	38.2–44.7
Poor education recall	278	13.4	11.4–15.7
Last time had diarrhea drank ORS	667	32.0	28.8–35.5
School factors (*N* = 116)			
Facilities (observed)			
Type of toilet			
Latrine	68	58.6	49.9–66.8
Septic tank	53	45.7	37.3–54.4
Pour flush	63	54.3	45.1–63.4
Number of toilets, median (IQR)	4 (2–6)	–	–
Number of handwash stations, median (IQR)	2 (1–3)	–	–
Students-to-toilet ratio, median (IQR)	52 (27–76)	–	–
Students-to-handwash station ratio, median (IQR)	76 (38–148)	–	–
Handwash station not near toilet	66	74.2	63.7–82.4
Provision of no separate toilets for female students	60	51.7	42.6–60.8
Insufficient soap available	83	71.6	62.8–78.9
Insufficient water available	30	25.9	18.7–34.6
Outcome			
Illness in past week (self-reported)			
Neither diarrhea nor vomiting	1,352	64.9	61.2–68.5
Diarrhea only	233	11.2	9.9–13.8
Vomiting only	253	12.1	10.3–14.3
Diarrhea and vomiting	244	11.7	9.2–13.5

CI = confidence interval; IQR = interquartile range; ORS = oral rehydration salts.

* Standard errors adjusted for school clustering and regional stratification.

**Table 3:** Unadjusted random effect multinomial regression models of self-reported diarrhea and vomiting among students and risk factors at Monastic schools across Myanmar (*N* = 2,082)

Factor	Diarrhea only (*N* = 244)			Vomiting only (*N* = 253)			Diarrhea and vomiting (*N* = 233)		
	*n* (%)	RR (95% CI)	*P* value	*n* (%)	RR (95% CI)	*P* value	*n* (%)	RR (95% CI)	*P* value
Student level									
Student characteristics									
Sex									
Male	129 (12.3)	1.14 (0.87–6–1.52)	0.37	134 (12.7)	1.14 (0.86–1.51)	0.35	122 (11.6)	1.11 (0.83–1.50)	0.46
Female	115 (11.2)	Ref.		119 (11.6)	Ref.		111 (10.8)	Ref.	
Grade									
4	128 (12.4)	1.41 (1.06–1.87)	0.02	146 (14.1)	1.74 (1.31–2.31)	< 0.01	125 (12.1)	1.48 (1.11–1.98)	0.01
5	116 (11.1)	Ref.		107 (10.2)	Ref.		108 (10.3)	Ref.	
Hygiene behavior									
Poor handwashing before meals									
Yes	53 (12.2)	1.09 (0.77–1.56)	0.62	70 (16.1)	1.51 (1.08–2.10)	0.02	64 (14.7)	1.49 (1.06–2.10)	0.02
No	191 (11.6)	Ref.		183 (11.1)	Ref.		169 (10.3)	Ref.	
Poor handwashing after toilet use									
Yes	52 (11.9)	1.02 (0.71–1.45)	0.93	61 (14.0)	1.19 (0.85–1.68)	0.31	59 (13.5)	1.27 (0.90–1.80)	0.18
No	192 (11.7)	Ref.		192 (11.7)	Ref.		174 (10.6)	Ref.	
Inconsistent toilet use									
Yes	115 (10.9)	1.00 (0.74–1.37)	0.98	139 (13.1)	1.37 (1.01–1.86)	0.04	133 (12.6)	1.50 (1.10–2.05)	0.01
No	129 (12.6)	Ref.		114 (11.2)	Ref.		100 (9.8)	Ref.	
Poor hygiene education recall									
Yes	32 (11.5)	0.92 (0.60–1.41)	0.71	31 (11.2)	0.85 (0.55–1.31)	0.47	38 (13.7)	1.19 (0.79–1.78)	0.40
No	212 (11.8)	Ref.		222 (12.3)	Ref.		195 (10.8)	Ref.	
School level									
Type of toilet									
Pour flush									
Yes	136 (11.4)	0.86 (0.56–1.31)	0.48	139 (11.6)	0.83 (0.54–1.27)	0.39	127 (10.6)	0.81 (0.53–1.25)	0.35
No	108 (12.2)	Ref.		114 (12.9)	Ref.		106 (12.0)	Ref.	
Latrine									
Yes	139 (11.7)	1.09 (0.70–1.70)	0.70	165 (13.8)	1.55 (0.99–2.42)	0.06	147 (12.3)	1.41 (0.90–2.21)	0.14
No	105 (11.8)	Ref.		88 (9.9)	Ref.		86 (9.7)	Ref.	
Septic tank									
Yes	101 (10.4)	0.70 (0.46–1.07)	0.77	110 (11.3)	0.77 (0.50–1.16)	0.87	88 (9.0)	0.60 (0.39–0.93)	0.39
No	143 (12.9)	Ref.		143 (12.9)	Ref.		145 (13.1)	Ref.	
Unclean toilets									
Yes	129 (11.2)	0.85 (0.54–1.34)	0.49	142 (12.3)	0.97 (0.62–1.52)	0.91	121 (10.5)	0.82 (0.52–1.29)	0.40
No	115 (12.4)	Ref.		111 (11.9)	Ref.		112 (12.0)	Ref.	
Number of toilets for students*									
1–2	73 (9.6)	0.65 (0.38–1.10)	0.11	109 (14.3)	1.26 (0.75–2.12)	0.39	90 (11.8)	0.84 (0.50–1.41)	0.51
3–5	74 (12.5)	0.80 (0.48–1.34)	0.40	69 (11.7)	0.96 (0.57–1.64)	0.90	50 (8.5)	0.56 (0.33–0.97)	0.04
6–20	97 (13.3)	Ref.		75 (10.3)	Ref.		93 (12.8)	Ref.	
No separate toilets for girls									
Yes	97 (10.1)	0.69 (0.45–1.07)	0.10	135 (14.0)	1.20 (0.78–1.85)	0.41	82 (8.5)	0.57 (0.37–0.89)	0.01
No	147 (13.1)	Ref.		118 (10.5)	Ref.		151 (13.5)	Ref.	
Handwashing facilities									
Insufficient soap available									
Yes	156 (10.5)	0.62 (0.40–0.97)	0.04	181 (12.2)	0.88 (0.56–1.39)	0.59	164 (11.0)	0.84 (0.53–1.32)	0.59
No	88 (14.8)	Ref.		72 (12.1)	Ref.		69 (11.6)	Ref.	
Insufficient water available									
Yes	42 (8.3)	0.62 (0.36–1.06)	0.08	56 (11.1)	0.84 (0.50–1.43)	0.52	72 (14.2)	1.33 (0.79–2.22)	0.28
No	202 (12.8)	Ref.		197 (12.5)	Ref.		161 (10.2)	Ref.	
Number of handwash stations*									
0–1	107 (12.3)	1.24 (0.72–2.16)	0.44	115 (13.2)	1.62 (0.92–2.84)	0.10	107 (12.3)	1.22 (0.70–2.12)	0.48
2–3	85 (11.5)	1.04 (0.59–1.85)	0.89	95 (12.9)	1.40 (0.79–2.52)	0.25	73 (9.9)	0.88 (0.49–1.57)	0.66
4–20	52 (11.0)	Ref.		43 (9.1)	Ref.	.	53 (11.2)	Ref.	
Unimproved water storage									
Yes	12 (14.8)	1.57 (0.67–3.64)	0.30	12 (14.8)	1.35 (0.57–3.17)	0.49	7 (8.6)	0.84 (0.32–2.21)	0.72
No	232 (11.6)	Ref.		241 (12.0)	Ref.		226 (11.3)	Ref.	

RR = risk ratio; CI = confidence interval.

* Groups represent sample tertiles.

**Table 4:** Adjusted random effect multinomial regression models for self-reported diarrhea and vomiting among students and risk factors at Monastic schools across Myanmar (*N* = 2,082)

Factor	Diarrhea only (*N* = 244)			Vomiting only (*N* = 253)			Diarrhea and vomiting (*N* = 233)		
	*n* (%)	ARR (95% CI)	*P* value	*n* (%)	ARR (95% CI)	*P* value	*n* (%)	ARR (95% CI)	*P* value
Student level									
Student characteristics									
Sex									
Male	129 (12.3)	1.16 (0.86–1.55)	0.33	134 (12.7)	1.13 (0.85–1.51)	0.40	122 (11.6)	1.12 (0.83–1.51)	0.46
Female	115 (11.2)	Ref.		119 (11.6)	Ref.		111 (10.8)	Ref.	
Grade									
4	128 (12.4)	1.40 (1.05–1.86)	0.02	146 (14.1)	1.75 (1.32–2.33)	< 0.01	125 (12.1)	1.48 (1.10–1.99)	0.01
5	116 (11.1)	Ref.		107 (10.2)	Ref.		108 (10.3)	Ref.	
Hygiene behavior									
Poor handwashing before meals									
Yes	53 (12.2)	1.06 (0.72–1.57)	0.76	70 (16.1)	1.43 (1.00–2.05)	0.05	64 (14.7)	1.31 (0.90–1.91)	0.16
No	191 (11.6)	Ref.		183 (11.1)	Ref.		169 (10.3)	Ref.	
Poor handwashing after toilet use									
Yes	52 (11.9)	0.99 (0.67–1.46)	0.95	61 (14.0)	0.98 (0.67–1.42)	0.91	59 (13.5)	1.14 (0.78–1.67)	0.51
No	192 (11.7)	Ref.		192 (11.7)	Ref.		174 (10.6)	Ref.	
Inconsistent toilet use									
Yes	115 (10.9)	1.08 (0.79–1.48)	0.64	139 (13.1)	1.31 (0.96–1.80)	0.09	133 (12.6)	1.55 (1.12–2.14)	0.01
No	129 (12.6)	Ref.		114 (11.2)	Ref.		100 (9.8)	Ref.	
Poor hygiene education recall									
Yes	32 (11.5)	0.95 (0.62–1.46)	0.81	31 (11.2)	0.81 (0.52–1.24)	0.33	38 (13.7)	1.10 (0.72–1.66)	0.68
No	212 (11.8)	Ref.		222 (12.3)	Ref.		195 (10.8)	Ref.	
School level									
Type of toilet									
Pour flush									
Yes	136 (11.4)	0.72 (0.44–1.17)	0.19	139 (11.6)	1.00 (0.62–1.61)	0.99	127 (10.6)	0.75 (0.46–1.23)	0.26
No	108 (12.2)	Ref.		114 (12.9)	Ref.		106 (12.0)	Ref.	
Latrine									
Yes	139 (11.7)	0.94 (0.60–1.49)	0.80	165 (13.8)	1.44 (0.91–2.28)	0.12	147 (12.3)	1.26 (0.79–2.02)	0.34
No	105 (11.8)	Ref.		88 (9.9)	Ref.		86 (9.7)	Ref.	
Septic tank									
Yes	101 (10.4)	0.61 (0.39–0.96)	0.03	110 (11.3)	0.80 (0.51–1.25)	0.33	88 (9.0)	0.66 (0.42–1.05)	0.08
No	143 (12.9)	Ref.		143 (12.9)	Ref.		145 (13.1)	Ref.	
Unclean toilets									
Yes	129 (11.2)	0.84 (0.54–1.32)	0.46	142 (12.3)	0.91 (0.58–1.42)	0.67	121 (10.5)	0.71 (0.45–1.13)	0.20
No	115 (12.4)	Ref.		111 (11.9)	Ref.		112 (12.0)	Ref.	
Number of toilets for students*									
1–2	73 (9.6)	0.68 (0.40–1.18)	0.17	109 (14.3)	1.17 (0.68–2.00)	0.58	90 (11.8)	0.91 (0.52–1.58)	0.73
3–5	74 (12.5)	0.74 (0.42–1.31)	0.31	69 (11.7)	0.90 (0.51–1.61)	0.73	50 (8.5)	0.49 (0.27–0.89)	0.01
6–20	97 (13.3)	Ref.		75 (10.3)	Ref.		93 (12.8)	Ref.	
No separate toilets for girls									
Yes	97 (10.1)	0.82 (0.50–1.34)	0.42	135 (14.0)	1.16 (0.70–1.90)	0.57	82 (8.5)	0.48 (0.28–0.82)	< 0.01
No	147 (13.1)	Ref.		118 (10.5)	Ref.		151 (13.5)	Ref.	
Handwashing facilities									
Insufficient soap available									
Yes	156 (10.5)	0.78 (0.48–1.28)	0.33	181 (12.2)	0.95 (0.57–1.57)	0.84	164 (11.0)	0.71 (0.42–1.19)	0.19
No	88 (14.8)	Ref.		72 (12.1)	Ref.		69 (11.6)	Ref.	
Insufficient water available									
Yes	42 (8.3)	0.45 (0.22–0.91)	0.03	56 (11.1)	0.56 (0.28–1.09)	0.09	72 (14.2)	1.47 (0.75–2.89)	0.26
No	202 (12.8)	Ref.		197 (12.5)	Ref.		161 (10.2)	Ref.	
Number of handwash stations*									
0–1	107 (12.3)	1.85 (0.98–3.49)	0.06	115 (13.2)	2.02 (1.05–3.87)	0.03	107 (12.3)	1.61 (0.84–3.09)	0.12
2–3	85 (11.5)	1.19 (0.66–2.14)	0.57	95 (12.9)	1.50 (0.82–2.76)	0.19	73 (9.9)	0.96 (0.52–1.75)	0.89
4–20	52 (11.0)	Ref.	–	43 (9.1)	Ref.	–	53 (11.2)	Ref.	–
Unimproved water storage									
Yes	12 (14.8)	2.61 (0.88–7.76)	0.09	12 (14.8)	1.78 (0.61–5.18)	0.29	7 (8.6)	0.69 (0.21–2.22)	0.53
No	232 (11.6)	Ref.		241 (12.0)	Ref.		226 (11.3)	Ref.	

ARR = adjusted risk ratio; CI = confidence interval.

* Groups represent sample tertiles.
