# Supplementary material for: Water, Sanitation, and Hygiene Facilities and Hygiene Practices Associated with Diarrhea and Vomiting in Monastic Schools, Myanmar
Source: Am J Trop Med Hyg. 2016 Aug 3;95(2):278–87. doi: 10.4269/ajtmh.15-0290 (PMC4973172; doi:10.4269/ajtmh.15-0290)
Supplement: Supplementary file 1 [file SD1.pdf]

Supplemental Table 1

Adjusted random effect multinomial regression models for self-reported diarrhea and vomiting among students and risk factors in Monastic schools in Myanmar using student-to-facility ratios to measure toilet and handwash station availability ($N = 1,659$)

Factor	Diarrhea only ($N = 204$)		Vomiting only ($N = 200$)		Diarrhea and vomiting ($N = 179$)	
	ARR (95% CI)	P value	ARR (95% CI)	P value	ARR (95% CI)	P value
Student level						
Student characteristics						
Sex						
Male	1.18 (0.86–1.62)	0.30	1.06 (0.77–1.46)	0.73	1.05 (0.75–1.48)	0.77
Female	Ref.		Ref.		Ref.	
Grade						
4	**1.46 (1.07–2.00)**	**0.02**	**2.13 (1.54–2.94)**	< **0.01**	**1.58 (1.13–2.21)**	**0.01**
5	**Ref.**		**Ref.**		**Ref.**	
Hygiene behavior						
Poor handwashing before meals						
Yes	1.01 (0.66–1.57)	0.95	1.06 (0.69–1.62)	**0.80**	1.34 (0.87–2.08)	0.185
No	Ref.		Ref.		Ref.	
Poor handwashing after toilet use						
Yes	1.01 (0.65–1.56)	0.98	1.24 (0.81–1.89)	0.32	1.03 (0.66–1.61)	0.91
No	Ref.		Ref.		Ref.	
Inconsistent toilet use						
Yes	1.07 (0.76–1.52)	0.70	1.34 (0.94–1.91)	0.11	1.37 (0.95–1.99)	0.09
No	Ref.		Ref.		Ref.	
Poor hygiene education recall						
Yes	0.91 (0.56–1.50)	0.72	0.96 (0.59–1.56)	0.88	1.17 (0.72–1.91)	0.52
No	Ref.		Ref.		Ref.	
School level						
Student-to-toilet ratio*						
< 51:1	Ref.		Ref.		Ref.	
51:1–100:1	**0.45 (0.26–0.78)**	< **0.01**	0.70 (0.40–1.20)	0.20	**0.49 (0.27–0.89)**	**0.02**
> 100:1	**0.48 (0.24–0.97)**	**0.04**	0.70 (0.35–1.40)	0.32	1.07 (0.52–2.18)	0.86
Type of toilet						
Pour flush						
Yes	0.71 (0.42–1.20)	0.20	0.87 (0.51–1.48)	0.61	0.74 (0.43–1.29)	0.29
No	Ref.		Ref.		Ref.	
Latrine						
Yes	0.90 (0.54–1.49)	0.68	1.16 (0.70–1.92)	0.56	1.29 (0.76–2.19)	0.35
No	Ref.		Ref.		Ref.	
Septic tank						
Yes	**0.59 (0.37–0.95)**	**0.03**	0.69 (0.43–1.19)	0.14	**0.58 (0.35–0.95)**	**0.03**
No	**Ref.**		Ref.		**Ref.**	
Unclean toilets						
Yes	0.72 (0.43–1.20)	0.72	0.82 (0.49–1.36)	0.44	0.60 (0.35–1.01)	0.05
No	Ref.		Ref.		Ref.	
No separate toilets for girls						
Yes	0.86 (0.51–1.45)	0.57	1.22 (0.72–2.05)	0.46	**0.45 (0.25–0.80)**	< **0.01**
No	Ref.		Ref.		**Ref.**	
Handwashing facilities						
Student-to-handwash station ratio†						
< 51:1	Ref.		Ref.		Ref.	
51:1–150:1	0.86 (0.48–1.53)	0.61	1.16 (0.65–2.06)	0.62	0.83 (0.46–1.50)	0.54
> 151:1	1.96 (1.02–3.75)	0.04	1.71 (0.89–3.30)	0.11	1.61 (0.81–3.18)	0.17
Insufficient soap available						
Yes	0.78 (0.48–1.27)	0.32	0.93 (0.57–1.52)	0.76	0.76 (0.46–1.26)	0.28
No	Ref.		Ref.		Ref.	
Insufficient water available						
Yes	0.48 (0.15–1.50)	0.21	0.41 (0.13–1.34)	0.14	2.47 (0.91–6.65)	0.08
No	Ref.		Ref.		Ref.	

ARR = adjusted relative risk ratio; CI = confidence interval.
*Student-to-toilet ratio = number of students at the school to the number of student toilets at the school.
†Student-to-handwash station ratio = number of students at the school to the number of handwash stations for students at the school.
